# Razor sharp: The role of Occam's razor in science

**DOI:** 10.1111/nyas.15086

**Published:** 2023-11-29

**Authors:** Johnjoe McFadden

**Affiliations:** ^1^ Leverhulme Quantum Biology Doctoral Training Centre University of Surrey Guildford UK

**Keywords:** bayesian inference, history of science, occam's razor, postmodernism, science education

## Abstract

Occam's razor—the principle of simplicity—has recently been attacked as a cultural bias without rational foundation. Increasingly, belief in pseudoscience and mysticism is growing. I argue that inclusion of Occam's razor is an essential factor that distinguishes science from superstition and pseudoscience. I also describe how the razor is embedded in Bayesian inference and argue that science is primarily the means to discover the simplest descriptions of our world.

Our modern world is built on the back of scientific advances such as modern electronics and modern molecular vaccinology that delivered protection from Covid‐19 disease within a year of the discovery of SARS‐CoV‐2. Yet, significant levels of distrust in the enterprise of science continue to be a feature of many cultures and societies. According to a recent Gallup poll, for example, 20% of Americans believe in the literal truth of the Christian bible, including the creation story.^1^ Covid‐19 vaccine hesitancy remains a persistent problem, with rates (as of 2021) as high as 45% (Russia), 46% (Italy), 43% (US), and 76% in Kuwait.^2^ A recent international survey asking people whether “they have a lot of trust in scientists to do what is right for the public” returned rates ranging from 14% to 60% in different countries, with a median of 36% and stark differences that depended on political leaning—for example, with only 20% of right‐identifying Americans trusting scientists compared to 62% of left‐identifying Americans.^3^ In education, despite progress there remains a considerable gender gap between achievement in STEM subjects of girls and women compared to boys and men at nearly all levels.^4^ Over 8% of patients in some countries opt for alternative or complementary treatments, such as homeopathic medicine, that are supported by public funds despite the fact that they undermine conventional treatments and are contrary to fundamental scientific principles such as the law of mass action (homeopathy) and have led to deaths.^5^, ^6^, ^7^, ^8^ Despite the absence of evidence of harm, GM crops such as Golden Rice, which is safe to eat^9^ and has the potential to prevent vitamin A deficiency (a leading cause of blindness and infant mortality in the world), continue to be banned in many countries, including those comprising the EU.^10^ Climate skepticism^a^ remains a significant challenge to implementation of climate control measures.^11^ A global survey of participants from 52 countries published in 2020 concluded that “public understanding of science is generally low”.^12^ In a recent article, philosophers Stefaan Blancke and Maarten Boudry attribute the prevalence of pseudoscience belief systems to several causes, prominent among them being a lack of understanding of fundamental principles of science.^13^

While the above is problematic, one can ask even scientists whether they fully understand the fundamental principles of their disciplines. Philosophy of science is rarely taught as a component of scientific education, but if pushed to identify the defining feature of their disciplines most scientists generally choose the *principle of falsifiability* (attributable to Karl Popper).^14^ Yet, attempting to falsify a theory or hypothesis can be as (or more) difficult as proving it's truth or veracity; this has been shown by re‐emergence of theories/hypotheses that had supposedly been falsified, such as inheritance of acquired characteristics, now accepted within epigenetics, or what Einstein called his “greatest blunder”, his cosmological constant,^15^ which has recently “remerged” as dark energy. Even in everyday practice, experimental scientists who discover evidence contrary to their favorite theory/hypothesis will often turn to “fudge factors” to accommodate contrary data.^16^

A famous example is the phlogiston theory invented by the German chemists/alchemists Georg Ernst Stahl (1659–1734) and Johann Joachim Becher (1635–82) to account for the observation that a wooden log loses mass when it is burned to ash. They named the material that leaves combustible material *phlogiston* (from the Greek *phlox* for flame) and claimed that it was the agent of heat and combustion. The later observation that some metals actually gain mass during burning might be assumed to have disproved the phlogiston theory, but to save the theory phlogiston advocates proposed that some forms possess negative mass.^17^ With an inexhaustible supply of fudge factors, even outlandish theories may remain consistent with any amount of experimental data. It should also be remembered that some theories are, in practice, non‐falsifiable (for example, string theory or the Big Bang) yet are generally accepted as *bone fide* components of the scientific enterprise.^18^

Other criteria that are often cited as essential for science do not define it. For example, experimentation is highlighted in many definitions of “science”; but the medieval alchemists performed thousands of experiments that got them nowhere. Moreover, chefs may experiment with a new recipe, just as a composer might experiment with new kinds of composition, but neither are considered to be doing science. And, as already highlighted, several areas of science, including pure mathematics and cosmology, are pursued without experimentation. Even one of the most famous scientific theories of the 19^th^ century, Darwin and Wallace's theory of evolution by natural selection, was not tested experimentally until the mid‐twentieth century, with experiments measuring the acquisition of genetic resistance to antibiotics by bacteria.

Another criterion frequently cited to be important for science is mathematical reasoning, but astrologers, numerologists, and homeopaths also make use of mathematical principles. Still other definitions emphasize science's systematic approach to knowledge acquisition. For example, according to Wikipedia, “Science is a systematic endeavor that builds and organizes knowledge in the form of testable explanations and predictions about the universe.”^19^ But the definition could easily be applied to almost any human endeavor, from cookery^b^ to plumbing or painting.

Without a guiding fundamental principle, the teaching of science tends to lurch between educational approaches that emphasize science as either a repository of knowledge or a methodology, prompting the educator Jonathan Osbourne to argue in response to proposed U.S. educational reforms that

a basic problem with the emphasis on teaching science through inquiry is that it represents a confusion of the goal of science—to discover new knowledge about the material world—with the goal of learning science—to build an understanding of the existing ideas that contemporary culture has built about the natural and living world that surround us.^20^

## The Razor

Occam's^c^ razor,^21^, ^22^ or the principle of parsimony that “entities should not be multiplied beyond necessity” was highlighted as a fundamental principle of modern science by many of its pioneers. The razor owes its name to the 14^th^ century Franciscan friar William of Occam (1287–1347).^21^ Born in the village of Ockham in Surrey, William studied and taught at Oxford where he used his razor and radical nominalism to dismantle much of medieval metaphysics, an accomplishment that led to his trial, before the Pope in Avignon, for heretical teaching. After accusing the Pope of heresy, William was forced to flee Avignon and died in exile in 1347. Yet his work inspired a new movement, known as the *via moderna*, in European universities that went on to influence the Renaissance, the Enlightenment, and the Scientific Revolution,^23^, ^24^, ^25^ though it is ignored in most histories of science today.

The earliest scientific applications of Occam's razor were to the heavens. The Parisian *via moderna* scholar Jean Buridan (1301–58) considered the motions of the heavens. To account for these, medieval astronomers preceding Buridan imported the ancient Greek complex system of crystal spheres that carried the stars, five visible planets, sun, and moon on their geocentric motions across the sky. For their diurnal rotations, Buridan considered a simpler model. He wrote that,

Just as it is better to save the appearances through fewer causes than through many . . .. Hence it is better to say that the earth (which is very small) is moved most rapidly and the highest sphere is at rest, than to say the opposite.^26^

Buridan was essentially arguing that the heavenly body's diurnal rotations could be just a matter of perspective from a rotating Earth and, in reality, “the highest sphere” carrying the fixed stars is stationary. Note that Buridan did not argue for a rotating Earth on any observational grounds but only that it should be *preferred* because it is a simpler model—hence, application of Occam's razor.

Despite Buridan's appeal to the razor, medieval astronomy remained dominated by the Ptolemaic geocentric model inherited from the Greek world, the latter accommodating the complex motions in the heavens with an equally complex model of circles within circles known as *epicycles*. Two centuries later, when Copernicus came to study the Ptolemaic model, he was horrified by several of its features prompting him to write that “having become aware of these defects, I often considered whether . . . it could be solved with fewer and much simpler constructions than were formerly used.”^27^ His radical solution was to allow the Earth to move first, like Buridan's model, by spinning on its axis each day. This move eliminated the Ptolemaic diurnal circles from the sun, moon, and planets, a simplification that helped Copernicus to discern an even simpler system with the Earth not only spinning but orbiting the sun each year, which eliminated Ptolemy's annual circles from each of the five visible planets.

Famously however, despite its heliocentric perspective, Copernicus’ system retained several features of Ptolemy's model, including perfect circles and uniform motion. Moreover, his heliocentric model made no more accurate predictions than his geocentric Greek predecessor. Lacking observational evidence in favor of heliocentricity, as Rhonda Martens has argued^28^ both Copernicus and his follower Rheticus justified his heliocentric model primarily on the grounds of its superior harmony and simplicity. For example, in his *Revolutions*, Copernicus argued that, “I hold it easier to concede this than to let the man be distracted by an almost endless multitude of circles, which those are obliged to do who detain [the earth] in the centre of the world.”^29^ Rheticus similarly claims that, “For in the common hypotheses [the Ptolemaic system] there appeared no end to the invention of spheres”.^30^

Despite the failure of Copernicus’ argument to provide more accurate predictions, the giants of the Scientific Revolution—from Tycho Brahe to Johannes Kepler, Galileo, and Newton—were convinced his heliocentric model should be preferred on the grounds that it was simpler. For example, Brahe claimed that heliocentricity “circumvents all that is superfluous and discordant in the system of Ptolemy”,^31^ whereas Kepler insisted that

She [nature] loves simplicity, she loves unity. Nothing ever exists in her which is useless or superfluous, but more often she uses one cause for many effects. Now under the customary [Ptolemaic] hypothesis there is no end to the invention of circles, but under Copernicus's a great many motions follow from a few circles.

In his *Dialogue Concerning the Two Chief World Systems*, published in 1632,^32^ Galileo sets out more detailed arguments. First the pro‐Copernican protagonist Salviati makes a case for preferring simpler solutions, writing that

it is much simpler and more natural to keep everything with single motion than to introduce two. But I do not assume that the introduction of the two be impossible, nor that I intend to draw a necessary proof of this; merely a greater probability‘‘ (*Dialogue* 2, second day).

It is interesting to note that, several centuries before Bayesian statistics aligned simplicity with probability (to be discussed below), Galileo had already recognized that the case for simplicity is based on probability rather than a proof. Salviati's protagonist in the *Dialogue*, Simplico, makes this explicit, as he points out to Salviati “it seems to me that you base your case on the greater ease and simplicity of producing the same effect”. Salviati goes on to apply the simplicity principle, first to fixed stars, arguing that “it seems to me that it is much more effective and convenient to make them immobile than to have them roam around ….”

In the third day of the *Dialogue*, Salviati discusses the planets arguing that “Ptolemy introduces vast epicycles adapting them one by one to each planet … all of which can be done away with by one simple motion of the Earth.” He also considers the absurd physicality of the Ptolemaic system asking,

do you not think it extremely absurd, Simplicio, that in Ptolemy's construction where all planets are assigned their own orbits, one above another, it should be necessary to say that Mars, placed above the sun's sphere, often falls so far that it breaks through the sun's orb, descends below this and gets closer to the earth than the body of the sun is, and then a little later soars immeasurably above it?

He goes to argue:

you see gentlemen, with what ease and simplicity the annual motions, if made by the Earth, lends itself for supplying reasons for the apparent anomalies which are observed in the movements of the five planets …. It removes them all and reduces these movements to equable and regular motion; and it is Nicolas Copernicus who first clarified for us the reason for this marvellous effect.

Finally, Sagredo, the arbiter of the debate, concludes:

for my part I am convinced, so far as my senses are concerned, there is a great difference in the simplicity and ease of effecting results by the means given in this new arrangement than the multiplicity, confusion and difficulty found in the ancient generally accepted one …. Thus it is said that Nature does not multiply things unnecessarily.

In his *Principles of Philosophy*, published in 1649,^33^ Descartes similarly identified the Copernican heliocentric system as ‘‘somewhat simpler and clearer’’ than either the Ptolemaic system or Brahe's geoheliocentric system in which the Earth remained at the center of the universe but the five planets revolved around the Sun, which itself orbits the Earth.

Isaac Newton did not explicitly make a case for the heliocentric system based on simplicity, presumably because by the time he wrote *The Mathematical Principles of Natural Philosophy* (also known simply as *The Principia*), in 1687, the heliocentric system was considered pretty much proven by the much greater accuracy of Kepler's astronomical predictions based on his heliocentric system. However, in *The Principia*, Newton nails his commitment to Occam's razor explicitly as Rule 1 in the section titled “Rules of Reasoning in Philosophy,” insisting that “we are to admit no more causes of natural things than such as are both true and sufficient to explain their appearances.”^34^ Newton's authority helped to cement the reputation of Occam's razor as a scientific virtue in modern science so that, for example, when describing the theory of natural selection, Alfred Russell Wallace (who independently of Darwin developed the theory of natural selection), wrote that “the theory itself is exceedingly simple, and the facts on which it rests—though excessively numerous individually, and coextensive with the entire organic world—yet come under a few simple and easily understood classes.”^35^

But not everyone was convinced of the value of simplicity. After publishing his special relativity equations in 1905, Albert Einstein strove to find relativistic laws that incorporated gravity and acceleration. His initial approach was to strive for completeness—by incorporating the maximal amount of data—rather than simplicity. He constructed equations that incorporated as many observations as possible and then attempted to work backwards to construct a simple unifying theory. He even berated his colleague Max Abraham for his alternative approach of first searching for the simplest or most elegant solutions, writing that “I was totally ‘bluffed’ by the beauty and simplicity of his [Abraham's] equations”.^36^ He went on to blame Abraham's failure on “what happens when one operates formally [looking for elegant mathematical solutions], without thinking physically.”

Yet, after spending around a decade ploughing, unsuccessfully, through one complex equation after another, Einstein eventually changed tack and adopted Abraham's approach of examining only the simplest and most elegant equations, and only later testing them against physical facts. This “razor‐first” approach led to the discovery of a theory Einstein described as “of incomparable beauty”, the general theory of relativity. This experience prompted him to re‐evaluate the role of simplicity in science and provided valuable insight into the usefulness of the razor in theory construction. He wrote that:

A theory can be tested by experience, but there is no way from experience to the construction of a theory, [adding that] equations of such complexity . . . can be found only through the discovery of a logically simple mathematical condition that determines the equations completely or almost completely.^36^

Here, Einstein describes what might be called the inverse problem of theory construction: that it is easy to start from a simple theory and generate complex outputs, but usually impossible to do the inverse. This insight is essentially equivalent to the well‐known category of inverse problems in physics, engineering, and mathematics: given some data or observations, the challenge is to find the parameters or inputs into a model that predicts those observations. The problem is that, although a precise configuration of inputs into a defined theory or model completely determines its outputs (predictions of the theory or model), the same is not true in reverse because the same set of data could have been generated by a wide, potentially infinite variety of theories or models that are suitably parameterized. So, the inverse problem has no unique solution (for example, a potential infinite combinations of electrical sources can generate the same EMF,^37^ a problem that gives rise to the difficulty in determining the precise electrical sources in the brain that gave rise to an observed EEG signal^38^, ^39^). The ancient problem of solving the motions of the heavenly bodies from Earth‐based observations was also an inverse problem, as demonstrated by the fact that two very different models—geocentric and heliocentric—generated very similar predictions.^d^ As David Merritt recently noted, “Even incorrect theories can make correct predictions, and there will always be an infinite number of theories (most of them yet undreamed of) that can correctly explain any finite set of observations.”^40^

## Backlash to the razor

Despite its success, the Occam's razor simplicity criterion favored by Copernicus and subsequent scientists has been criticized by several prominent historians of science, including Thomas Kuhn^41^ and Arthur Koestler.^42^ They argue instead that application of the razor is based largely on ascetic, philosophical or cultural, rather than scientific, grounds. In his book, *The Sleepwalkers: A History of Man's Changing Vision of the Universe* Koestler points out that because Copernicus retained perfect circles and uniform motion in his model, he had to introduce additional epicycles so that his final circle count was like Ptolemy's. Similarly, although in *The Structure of Scientific Revolutions* ^41^ Kuhn listed simplicity among his five core theoretical virtues that characterize science, he went on to insist that “[j]udged on purely practical grounds, Copernicus’ new planetary system was a failure, it was neither more accurate nor significantly simpler than its Ptolemaic predecessor.” He also claimed that Copernicus’ arguments from harmony or unification “appeal, if at all … [to an] aesthetic sense, and that alone”.^43^ Lacking justification in either simplicity (one of five core theoretical virtues that characterize science) or accuracy, Kuhn concluded that scientific advances, such as the Copernican revolution, are based not solely on reason, but also on cultural bias, irrationality, and aesthetic preference.

This criticism of the influence of Occam's razor in science remains common. In medical diagnosis the razor is sometimes contrasted with *Hickam's dictum*, which states that patients can have more than one disease rather than a single cause of their symptoms.^e^ In the scientific literature, Occam's razor has been attacked with claims that “its rhetorical purpose [is] as an old saw persuading us to champion the supposed virtue of simplicity.”^44^ In systems biology, for example, it has been claimed that because life is “irreducibly complex” Occam's razor has no role in model selection.^45^ Recent popular science articles by influential authors have made similar claims that echo Kuhn and Koestler's dismissal of Copernicus’ simplicity claim, arguing that Occam's razor represents the “tyranny of simple explanations”^46^ or that it is “appealing, widely believed, and deeply misleading”.^47^ This dismissal of simplicity as a criterion for scientific advance has likely contributed to what has been described as “a worrying trend to favour unnecessarily complex interpretations”.^48^

The dismissal of simplicity as a valid criterion in science has, unsurprisingly, been picked up by critics of the scientific endeavor including post‐modernist and relativist philosophers who insist that, without it, science has no more claim to objective truth than witchcraft, folk belief, or astrology. For example, the philosopher of science Paul Feyerabend argued that:

To those who look at the rich material provided by history [of science] . . . it will become clear that there is only one principle that can be defended under all circumstances and in all stages of human development. It is the principle: anything goes.^49^

According to postmodernists such as Feyerabend, science merely takes its place alongside other belief systems such as religion, mysticism, witchcraft, folk beliefs, astrology, homeopathy, or the paranormal. Each has, they claim, its own truths and none can claim any monopoly on the truth. Feyerabend went on to champion his relativistic philosophy in public education arguing that science should not have any privileged status in school classes over mysticism, magic, or religion, a view that was enthusiastically adopted by a generation of relativist and constructivist philosophers and educators^50^ and purveyors of so‐called creation science.^51^ The supposed failure of the principle of simplicity to account for the success of Copernicus’ heliocentric model of the solar system has been, and remains, an influential idea. But is the criticism valid?

## Was Copernicus’ heliocentric system simpler than Ptolemy's geocentric model?

The key point is that neither Copernicus nor his followers such as Brahe, Kepler, Galileo, or Newton based their support for the supposed simplicity of his heliocentric model on any kind of crude circle count. Instead, their case was based on the recognition of features that made no physical sense in the geocentric system but were accommodated by additional complexity that could be removed in the heliocentric system. For example, in the phenomenon known as retrograde motion, planets, which normally travel from East to West across the sky, occasionally reverse their direction of motion to travel West to East, before reversing again. In Ptolemy's model, retrograde motion is accommodated with planetary epicycles—essentially a wheel within a wheel—but never explained. In the heliocentric model, these epicycles can be removed as retrograde motion is revealed as an effect of perspective, caused by the Earth either overtaking, or being overtaken by, the retrograde planet as both orbit the Sun.

Another set of planetary epicycles in the Ptolemaic model has a period that exactly matches the Earth year. What is an Earth year doing in the geocentric orbit of, say, Jupiter or Mars? It makes no physical sense. The annual periodic motion is, once again, accommodated in Ptolemy's model by an epicycle, but not explained. These epicycles vanish in Copernicus’ model once its frame of reference is shifted from the Earth to the sun.

It was the heliocentric system's elimination of arbitrary and physically infeasible features, such as these, rather any circle count, that convinced the giants of modern science that Copernicus's system was simpler, and thereby more likely to be right. As Tycho Brahe had insisted, heliocentricity does indeed “circumvents all that is superfluous and discordant in the system of Ptolemy.”^31^ However, as Koestler, Kuhn and others point out, because he retained both perfect circles and uniform motion, Copernicus did indeed have to add additional epicycles that corrected for the eccentricity of the planetary orbits and variations in orbital velocity. But, as Galileo had pointed out four centuries earlier, these are tiny compared to the “vast epicycles” needed in the geocentric system that (unknowingly) corrected for the Earth's rotation and orbit around the sun.

**FIGURE 1:**
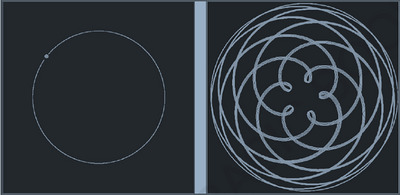
Plot of orbit of the planet Venus from heliocentric (left) and geocentric perspective with the semi‐major axis earth equal to 0.0167 which is about the linear eccentricity of the planet's orbit. Drawn with Gerd Breitenbach's “Curves of planetary motion in geocentric perspective” orbital simulator available at http://gerdbreitenbach.de/planet/planet.html.

For example, Venus’ orbit has an eccentricity of 0.0068, which is very close to being a perfect circle. The difference between the two corrections is apparent in Figure 1, where the orbit of Venus (with 0.0068 eccentricity) has been plotted from a heliocentric (left) and geocentric (right) perspective. The deviation from perfect circularity is there in the heliocentric model but it is tiny compared to the geocentric model. Kepler did not need to count circles to recognize which model was simpler and thereby provided a suitable platform for his revolutionary move of bending the circles into (slight) ellipses and abandoning Copernicus’ commitment to uniform motion, to reveal the simple model of the solar system that we know today.

So the giants of modern science were not fooled by Copernicus’ claim that his model was simpler than Ptolemy's. As a physical model of the world, rather than a calculating device, it was indeed simpler. But are simpler models more likely to be “true”?

## The Bayesian razor

As Elliot Sober has argued, there is not one but many forms of Occam's razor,^21^ each with its own meaning and justification. However, the form of the razor that is embedded (though often not recognized as such) in modern science can be called the Bayesian razor.

Although Bayesian inference was developed more than two centuries ago, it wasn't until 1939 that the statistician and geophysicist Harold Jeffreys provided a quantitative form of Occam's razor via Bayesian statistics in Chapter 5 of the first edition of his 1939 textbook *Theory of Probability*.^52^ Further contributions were made by William Jefferys and James Berger,^53^, ^54^ as well as David MacKay^55^ in the early 1990's. Their insights can be illustrated with the assistance of two dice: a simple six‐sided die and a more complex 60‐sided die. Say I have both dice and hidden I throw one of them. I call out the number 39 and ask you to guess which die I have thrown. You consult the Bayes' equation *P*(*A*|*B*) = *P*(*B*|*A*) × *P*(*A*)/*P*(*B*), where *A* and *B* are events, such as throwing either a six or sixty‐sided die (the hypothesis) or that that a die lands on a particular number (the data). *P* is probability. So, *P*(*A*) is the *prior probability* of throwing either the six or sixty‐sided die which, assuming I am fair, is 0.5 for each. *P*(*B*) is a normalizing factor that can be ignored in this example. From the perspective of Occam's razor, the key factor is *P*(*B*|*A*), which is known as the *likelihood*, that is, the probability that the data would have been generated, given the hypothesis *P*(*A*|*B*). The value we want to calculate in this example is the *posterior probability* of both dice having thrown the number 39. For the six‐sided hypothesis, the likelihood, *P*(*B*|*A*) is equal to zero, since a six‐sided die cannot possibly throw the number 39. Multiplying the prior probability of 0.5 by zero gives us zero; so there is zero probability that I threw the six‐sided die. For the sixty‐sided die hypothesis, the likelihood of a sixty‐sided die throwing the number 39, *P*(*B*|*A*) equals 1/60. Multiplying this by the prior of 0.5 gives a posterior probability of 1/120. To compare these two hypotheses, we divide the larger by the smaller posterior probability, in this case 1/120 divided by zero, which equals infinity. The sixty‐sided die is infinitely more likely to have been the source of the data than the six‐sided die.

The answer is, of course, obvious without having to resort to Bayesian inference. But it is less obvious for another example. Instead of, say, the number 39 after throwing one of the two unseen dice again, I call out the number 5. This number could have been generated by a throw of either die. Since they have the same prior probability, are both dice equally likely? Both Occam's razor and Bayesian inference insist that the simpler hypothesis, the six‐sided die, should be preferred. This can be seen is we go through the sums again. The priors of 0.5 are unchanged and the likelihood of the sixty‐sided die throwing the number five is the same as it was for throwing the number 39, 1/60. But whereas there was zero likelihood of the six‐sided die throwing the number 39, there is a 1/6 likelihood of it throwing the number 5. When these likelihoods are multiplied by the 0.5 priors, then the posterior probability for the sixty‐sided die throwing a 5 is again 1/120 but the posterior probability for the six‐sided die throwing the same number is now 1/12. Comparing the two hypotheses, the simpler six‐sided die is ten times more likely to have been the source of the data than the sixty‐sided die. Occam's razor and Bayesian inference agree that, all things being equal, we should choose the simpler hypothesis.

Note however, that Bayesian razor delivers probabilities, not certainty (just as Galileo had intuited several centuries earlier). Sixty‐sided dice do occasionally throw the number 5, just as complex explanations are sometimes correct. But if both simple and complex models, theories, or hypotheses account for the data equally well—just as did the Ptolemaic and Copernican models—then, according to both Occam's razor and Bayesian inference, the simplest is more likely to be the source of that data.

A useful way of visualizing the Bayesian razor is as a comparison between the virtual space of possible data and the space of the actual data. In the dice example, the space of actual data is the same for both hypotheses: it is a single number. The space of possible data is, however, ten‐times larger for the more complex sixty‐sided die compared to the six‐sided die. The Bayesian razor is then a measure of the degree of collapse of the space of the possible data onto the space of the data. Simple models make sharp predictions, so the collapse is small. They are favored over complex models with many parameters and larger possible data space that can be adjusted to fit a wider range of data. As the physicist John von Neuman famously quipped “with four parameters I can fit an elephant, and with five I can make him wiggle his trunk”.^56^ In this sense, Ptolemy's model was an astronomical elephant that, with its ready availability of huge epicycles, could have fitted almost any kind of motions in the heavens. The Bayesian likelihood of the data, given the multiplicity of possible models, was small. Although Copernicus's model possessed a similar number of epicycles, most were tiny compared to Ptolemy's so the space of possible data that they could be fitted to them was much more constrained. The Bayesian likelihood of the data given the model was much larger than Ptolemy's. Models with even fewer parameters, such as Kepler's elliptical solar system, make even sharper predictions, so that, in a sense, it would be a miracle if they fitted the data if they don't also happen to be true.

The giants of modern science—such as Kepler, Galileo, or Newton—did not need Bayesian inference to know that Copernicus’ model was simpler than Ptolemy's. As modern scientists, they had an instinctive regard and respect for simplicity.^f^ Karl Popper—a favorite philosopher of many contemporary scientists and widely considered a giant of philosophy of science—argued that simplicity goes together with the *criterion of falsifiability*. In *The Logic of Scientific Discovery* Popper wrote that

Above all, our theory explains why simplicity *is so desirable*. To understand this there is no need to assume ‘a principle of economy of thought’ or anything of this kind. Simple statements, if knowledge is our object, are to be prized more highly than less simple ones *because they tell us more; because their empirical content is greater; and because they are better testable*. [Popper's italics].^14^

Simple theories make sharper predictions than complex theories, and so can be disproved by a greater range of data.

## Occam's razor and biology

But when is it justified to opt for a more complex model? This can be explored in the field of biology where the role of the razor is most often challenged with claims that biological systems are irreducibly complex in the sense that they cannot be reduced to a collection of parts. With arguments such as these, the biochemist and popular science writer, Michael Behe, went on to argue that this irreducibility could not be accounted for in standard neo‐Darwinian evolutionary theory.^57^ Although this claim has been widely refuted in the biological literature (see e.g. Ref. ^58^), it does highlight the danger of accepting that there are areas of science where Occam's razor is inappropriate.

One of the areas where the role of Occam's razor remains particularly controversial is its application to complex systems, such as in the science of systems biology. Model construction is central to systems biology but there remains a tension between the aims of the modeler for completeness and the use of Bayesian approaches for model selection that automatically incorporate a preference for simple models.

For example, genome‐scale modelling of phenomena such as metabolism or intracellular signaling, tends to aim for completeness by including as many enzymatic steps, genes, regulators, and pathways that can be deduced from genomes. However, the problem of fitting, for example, metabolic flux values, to networks, scales with the exponential of the size of the network, so it becomes computationally intractable for genome‐scale networks with potentially many hundreds or thousands of enzymatic steps. For this reason, techniques, such as ^13^C‐metabolic flux analysis (MFA),^59^, ^60^ use only relatively small‐scale networks of up to 100 or so reactions, representing sub‐pathways such as central metabolism, that can parameterized with data, such as mass spectrometry‐derived isotopomer fractions. Even these networks are, typically, underdetermined, so there are many, usually, millions, of possible flux solutions that need to explored with methods such as genetic algorithms, Monte Carlo simulations or simulated annealing,^61^ to find the optimal set of parameter values that best fits the data. There will usually be many possible “best fit” solutions that range from the simplest to much more complex. What criterion should be used to sort them? One of the pioneers of systems biology, Fridolin Gross advised against the use of Occam's razor, writing that “systems biology offers formal models as a remedy for such inferential failures, but the kind of simplicity introduced in the models drives them away from the truth”.^62^

**FIGURE 2:**
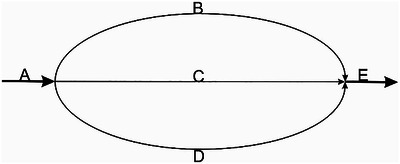
Hypothetical metabolic pathway where metabolite A is converted to metabolite E via any combination of pathways B, C, or D.

Consider a trifurcating step in a metabolic pathway in a microorganism such as is illustrated in Figure 2 where one mole of metabolite A is converted to one mole of metabolite E via three alternative metabolic intermediates, B, C, or D mediated by three different enzymatic reactions in pathways B, C, or D. The task is to determine the actual flux through the network. Methods, such as ^13^C‐MFA, may be applied to determine the parameter values for fluxes B, C, and D that best fit the data. Clearly, the simplest solution has only a single flux with normalized value 1 through B, C, or D as this would generate the same data as the situation where only a single pathway existed, and one pathway is clearly simpler than three. Let's imagine that the simplest solution is with flux only going through B. However, data are inherently noisy, so not all the data will be accounted for by a single flux solution. The experiment is performed, and the data are fitted and both maximal likelihood and Bayesian methods are used to fit the noisy data to the model. A typical result from Bayesian methods that incorporate Occam's razor and thereby favor the simplest solutions would be the predict that all the flux goes through pathway B, since one flux is simpler that two fluxes. Maximal likelihood methods that favor fit of the data to a model would likely predict a more complex solution in which, say, 90% of flux goes through pathway B with about 5% of flux going through each of C and D. Gross's advice would be to avoid the Bayesian approaches with their preference for simple solution that, he warned, drive the scientist further from the “truth”.

But what is “truth” in model fitting? Gross’ advice implies that we somehow know the truth prior to the experiment. In a sense, we do, in that the genome sequence, transcriptomics, and proteomics data may all agree that pathways C and D are encoded in the genome of the imaginary organism. If science is about discovering truth, then surely all three pathways should be included in any solution.

A key insight into the nature of science was made eight centuries ago by William of Occam, who wrote that:

the science of nature is neither about the things that are born and die, nor about natural substances, nor about the things we see moving around …. Properly speaking, the science of nature is about intentions of the mind that are common to such things, and that stand precisely for such things in many statements.^63^

In this extraordinary statement, William was, I believe, pointing out that science isn't strictly about truth in the sense of attaining complete knowledge of the nature of real objects in the world, because, ultimately, there is no way to (as one might say) “peek behind the veil of our sensory perceptions” to confirm that models derived from sensory perceptions and data correspond to a truth in the world. Instead, science is about constructing mental models or theories that, once generated, make psychological sense of the sensory data generated by ultimately unknowable objects in the world.

Note however that, although, as a nominalist, William opposed the prevailing medieval notion of philosophical realism, that abstract qualities are real existing things existing out there in the world, he was not an anti‐realist in the modern sense of denying that relationships between objects are extra‐mental. For William “the intellect does nothing to bring it about that the universe is one, or that a whole is composed [of its parts], or that … a triangle has three [angles]”.^64^ So, objects, such as triangles, and the relations between them, are for William real existing things in the world.

In my view, the point William was making with his “intentions of the mind” statement is that science is about constructing mental models with terms, such as, for example, “mass”, that are “common to such things” in the sense that they represent a real property of real objects that “that stand precisely for such things in many statements”—such as the law of mass action or Newton's laws of motion that allow many statements to be made about such as about the rate of fall of apples or planets. But William also realized that if a scientific theory (but not the objects it aims to describe) is just a mental construct, then there is no limit to the number of (mental) entities that can be included. To avoid the pitfall of “over‐parameterized” models (i.e., having entities beyond necessity, such as angels), William proposed that science should adopt the principle of simplicity—his eponymous razor.

Adopting this perspective, a Bayesian solution that favors a single pathway from A to E is not making an ontological claim about truth in the world but an epistemological claim that there is no evidence from the data for the operation of pathways ABE or ADE, so it should be removed from the model that generated the data.

The question arises then of when is it justified to include reactions B and D in the model? The answer is: when there is evidence for their activity in the data; for example, when metabolites are detected that are only present in those pathways. Importantly, this doesn't mean that one simply abandons Occam's razor. Instead, the additional data are incorporated into Bayes’ equation that, like the arrival of the number 39 in the dice example, will then output “zero probabilities” for solutions that do not include reactions B and D.

The alternative approach of adopting more complex solutions on the basis of prior knowledge of what is true is likely to result in errors, such as fitting experimental noise to inactive model pathways and thereby delivering the systems biology equivalent of von Neumann's elephant. Another important advantage of using simpler models in biology is that noise in the data remains as such (noise), rather than being overfitted to parameters. Experimental and theoretical studies have demonstrated an important role for noise in biological systems where, for example, it can give rise to control properties of metabolic systems.^65^, ^66^ Fitting noise to deterministic models may overlook a functional role for noise in biological systems, thereby generating erroneous conclusions.

## CONCLUSIONS

To appreciate the value of Occam's razor, it is instructive to follow the career of Robert Boyle, often hailed as the father of modern chemistry, who began his chemical career as a mystic and alchemist. He built a laboratory at Stalbridge where he indulged his passion for experiments that followed obscure formulae with exotic ingredients and instructions such as “alter and dissolve the sea and the woman between winter and spring;” he also wrote excitedly about a worm on the “Sombrero Coast” that transforms first “into a tree or then into a stone.” He related tales of a “foreign chemist” who claimed to have met a tonsured monk who could summon wolves out of thin air.^67^ Although this seems all very bizarre and nonsensical today, many of the greatest intellects of the Enlightenment (including, though later than Boyle, Newton and Johann Joachim Becher (who developed the phlogiston theory of combustion)) were adherents of alchemy. Why did chemistry flourish whereas alchemy died, while both utilized mathematics, experiment, and other tools of science? In *The Sceptical Chymist: or Chymico‐Physical Doubts & Paradoxes*, published in 1661, the mature Boyle provides us with his own reasoning, arguing against

noble Experiments, Theories, which either like Peacock's feathers made a great show, but are neither solid nor useful, or else like Apes, if they have some appearance of being rational, are blemished with some absurdity or other, that when they are Attentively consider'd, makes them appear Ridiculous.^68^

Boyle recognized that the greatest challenge for 17^th^ century scientists was to identify those theories that give rise to solid and useful science. He provided ten key principles by which “good and excellent hypotheses” could be separated from the “peacock theories”. Around half are based, in one way or another, on the principle of simplicity. For example, the seventh principle asserts that good theories should “clearly intelligible be”,^22^ a mark of simple theories. His sixth principle is more explicit stating that “a great part of the work of true philosophers has been, to reduce the true principles of things to the smallest number they can, without making them insufficient.” Boyle doesn't identify who the true philosophers were, but in another passage he refers “to the generally owned rule about hypotheses, that *entia non sunt multiplicanda absque necessitate”* (“entities must not be multiplied beyond necessity,” i.e., Occam's razor).^22^, ^69^ As already noted, Newton included the razor in his principles of science so that despite being a believer he excluded alchemy or other mystical notions from his greatest scientific work, *The Principia*.

The medieval world and the Middle Ages were beset with esoteric theories filled with angels, demons, gods, fabulous animals, mystical influences, and both benign and malign spirits that could transform base metals into gold, worms into trees, or invent fish that could sink ships.^70^ By applying Occam's razor, the giants of the Enlightenment and the Scientific Revolution rejected entities beyond necessity—and thereby mysticism, religion, and theology—to forge modern science. Our world today is seemingly beset with belief in theories of divine, mystical, and supernatural forces, as well as peacock theories that are more likely referred to as “pseudoscience” and “fake news.” We should, following the giants of modern science, keep Occam's razor close when practicing or teaching science, not least because simple theories are more easily communicated and understood. The message that science is, ultimately, the method by which we use the tools of experimentation, mathematics, and logic to find the simplest explanations of the complex phenomena of our world provides a clear mission statement for the whole of the scientific enterprise.

## AUTHOR CONTRIBUTIONS

JJMcF was responsible for conceptualization, writing, revising and editing.

## COMPETING INTERESTS

The author declares no competing interests.

### PEER REVIEW

The peer review history for this article is available at https://publons.com/publon/10.1111/nyas.15086

## Footnotes

a Often supported by populist politicians, who appear to be on the ascendency across the globe.

b E.g., cookery is a systematic endeavor that builds and organizes knowledge of flavors, foods, and cooking procedures to form testable predictions of what makes a tasty meal.

c The alternative spelling “Ockham” is used in many publications.

d Copernicus's revolutionary move can be seen as an early example of Einstein's later approach of finding a simple model or equation and only later checking whether it fits the facts. Copernicus was lucky, his heliocentric model did pretty well. Simple theories or models are not guaranteed to be *true* or even the best models, but they are a good starting point as there are vastly fewer simple than complex models.

e One form of Hickam's dictum states: “A man can have as many diseases as he damn well pleases.” See “Letter from the editor: Occam versus Hickam”. https://doi.org/10.1016/S0037‐198X(98)80001‐1

f Tall tales tend to be long tales, and they knew that it would take a very long tale to provide a physical model that was consistent with the Ptolemaic system.
